# Supplementary material for: Deliberate practice for retinopathy of prematurity: Retinal laser training using schematic eyes in ophthalmology education
Source: PLoS One. 2025 May 29;20(5):e0323365. doi: 10.1371/journal.pone.0323365 (PMC12121759; doi:10.1371/journal.pone.0323365)
Supplement: S1 File — (DOCX) [file pone.0323365.s001.docx]

#### Institution: (1) PSU (2) KKU (3) CMU **ID:** ___ ___

# Evaluation Form After Watching the Video

# Laser Indirect Ophthalmoscopy (LIO) for Retinopathy of Prematurity (ROP)

# Which of the following statements about laser radiation safety for LIO in ROP is incorrect?

1. Press treats at all times.
2. Press stands by when not firing the laser.
3. Do not turn your face or look up while the laser is active.
4. Recommend that the assistant wear laser-protective glasses.
5. Which of the following statements about using safety goggles for LIO in ROP is incorrect?
6. Provide safety goggles for all staff.
7. Provide laser-protective goggles suitable for the laser type used for all staff.
8. Provide laser-protective goggles for the assistant only.
9. Provide laser-protective goggles for the assistant only, and instruct other staff to exercise caution.
10. Which of the following statements about using lenses for LIO in ROP is incorrect?
11. Use any lens suitable for fundus examination.
12. Use a 28 D lens.
13. Position the lens front facing the patient's eye with the letters upright.
14. Continuously adjust the lens to ensure it remains perpendicular to the laser and retina during the procedure.
15. Which of the following statements about verifying the eye and retinal treatment area for LIO in ROP is incorrect?
16. Check and laser simultaneously.
17. Mark the eyes and inspect the retina area to be treated before performing the procedure.
18. Regularly verify the accuracy of laser’s positioning.
19. Verify the eye to be treated before firing the laser.
20. Which of the following statements about dilating pupils for LIO in ROP is incorrect?
    1. Prescribe pupil dilation with the available drugs (Mydriacyl + 10% Phenylephrine) before the procedure.
    2. Prescribe pupil dilation with drugs appropriate for infants (Mydriacyl + 1–2.5% Phenylephrine).
    3. Avoid dropping the medication directly onto the cornea.
    4. Monitor vital signs during pupil dilation.

## 6. Which of the following statements about using an eye speculum for LIO in ROP is incorrect?

1. Use the smallest size possible.
2. Use a large enough size to ensure the child's eye remains adequately open.
3. Use a model without eyelash guards.
4. Use a model that allows for rotating or pressing the eye in all peripheral areas.

## 7. Which of the following statements about choosing indentation for LIO in ROP is incorrect?

1. Use small cotton-tipped swabs for wide areas of the eye.
2. Use cleaned paper clips for narrow areas, with the tip facing toward the eyeball.
3. Use cleaned paper clips for narrow areas, with the tip facing away from the eyeball.
4. Use retinal indentation for areas that are not excessively peripheral.

## 8. Which of the following statements about selecting LIO cable spot size for LIO in ROP is incorrect?

1. Use any laser cable capable of firing a laser.
2. The laser size depends on the type of LIO cable used.
3. The laser size depends on the distance between the laser and the retina.
4. Both small and large spot sizes are available for LIO cables.

## 9. Which of the following statements about laser settings (Power, Duration, Interval) for LIO in ROP is incorrect?

- 1. For small spot size cables, start with settings: 200, 200, 400.
  2. For large spot size cables, start with settings: 300, 300, 400.
  3. For both cable types, start with settings: 900, 900, 900.
  4. Adjust laser power until a grayish-white burn is visible.

## 10. Which of the following statements about laser testing and lining for LIO in ROP is incorrect?

1. Start the laser from the ora toward the ridge.
2. Start the laser from near the ridge toward the ora.
3. Test the laser power in the avascular area before continuous firing.
4. Adjust the laser power to produce grayish-white marks, adjusting as needed for different areas.

## 11. Which of the following statements about laser spot placement and distribution for LIO in ROP is correct?

1. The distance between laser spots should be edge-to-edge overlapping.
2. The distance between laser spots should be slightly apart.
3. The distance between laser spots should correspond to the skipping of one spot.
4. Laser should target avascular areas overlapping the ridge.

## 12. Which of the following statements about adequate laser spot coverage for LIO in ROP is incorrect?

1. Cover the avascular area.
2. Overlap the retinal ridge.
3. Place spots close to the ora.
4. Place spots close to the retinal ridge.

## 13. Which of the following statements about corneal care for LIO in ROP is incorrect?

1. Avoid prolonged pressure on the eye.
2. Avoid excessive eye movement.
3. Regularly apply balanced salt solution (BSS) drops to the cornea during laser treatment.
4. Check for corneal abrasions after the procedure.

## 14. Which of the following equipment for LIO in ROP is/are necessary?

- 1. Scleral indentation.
  2. Eye speculum.
  3. 28 D lens.
  4. All of the above.

## 15. Which of the following statements about procedure flow for LIO in ROP is correct?

1. Examination → pupil dilation → general anesthesia → retinal exam → laser setup → laser treatment → macular safety check.
2. General anesthesia → retinal exam → laser setup → laser treatment → macular safety check.
3. General anesthesia → pupil dilation → retinal exam → laser setup → laser treatment → macular safety check.
4. General anesthesia → pupil dilation → laser setup → laser treatment → macular safety check.

## 16. Which of the following statements about maintaining laser focus during LIO in ROP is correct?

1. Continuous focus on the retina is unnecessary during laser treatment.
2. Maintain focus on the retina throughout the laser treatment.
3. Maintain focus on the retina for more than 50% of the laser treatment time.
4. Maintain focus on the retina for more than 80% of the laser treatment time.

## 17. Which of the following statements about laser setting adjustments during LIO in ROP is correct?

1. Use default laser settings throughout the procedure.
2. Adjust laser intensity appropriately during the procedure.
3. If the laser mark appears dark gray, reduce the laser intensity.
4. If the laser mark appears white, increase the laser intensity.

## 18. Which of the following statements about areas to avoid during LIO in ROP is incorrect?

1. Cornea.
2. Lens.
3. Macula.
4. Avascular retina.

## Comments or Suggestions Before Performing LIO in ROP

- ..............................................................................................................................

- ..............................................................................................................................

- ..............................................................................................................................
