# Supplementary material for: Deliberate practice for retinopathy of prematurity: Retinal laser training using schematic eyes in ophthalmology education
Source: PLoS One. 2025 May 29;20(5):e0323365. doi: 10.1371/journal.pone.0323365 (PMC12121759; doi:10.1371/journal.pone.0323365)
Supplement: S2 File — (DOCX) [file pone.0323365.s002.docx]

#### Rubric for Evaluating the LIO Procedure

#### Institution: (1) PSU (2) KKU (3) CMU **ID:** ___ ___

#### **Date:** ........../............../............... (DD/MM/YYYY) Pre-test/Post-test **Evaluator ID:** AJ___ ___

| **No.** | **Steps of LIO** | **Novice**  **(Score = 1)** | **Beginner**  **(Score = 2)** | **Advanced Beginner**  **(Score = 3)** | **Competent**  **(Score = 4)** | **Not Applicable**  **(Score = 0)** |
| --- | --- | --- | --- | --- | --- | --- |
| 1 | **Laser Radiation Safety** | Leaves the laser on even when not in use or fails to take precautions when raising their face during laser exposure. | Occasionally uses standby mode when not in use and takes some precautions when raising their face during laser exposure. | Consistently uses standby mode and takes proper precautions when raising their face during laser exposure. | Always uses standby mode and takes full precautions during laser exposure. |  |
| 2 | **Safety Goggle Use** | Fails to provide goggles for all relevant personnel. | Provides goggles for assistants but not all relevant personnel; does not warn others. | Provides goggles for assistants only and warns others. | Appropriately provides goggles for all relevant personnel. |  |
| 3 | **Lens Handling** | Selects an inappropriate lens or holds it incorrectly. | Selects the correct lens and orientation; provides mostly clear visibility. | Selects the correct lens and orientation; ensures clear and comprehensive retinal visibility. | Selects the correct lens and orientation; ensures optimal visibility with positional adjustments as needed. |  |
| 4 | **Site Verification** | Fails to ensure the laser is targeting the correct eye. | Ensures correct eye targeting; marks the site. | Ensures correct eye targeting, marks, and checks before treatment. | Ensures correct eye targeting, marks, and continuously verifies throughout treatment. |  |
| 5 | **Pupil Dilation** | Fails to take precautions during pupil dilation. | Dilates pupils effectively. | Dilates pupils effectively with consideration of potential corneal side effects. | Dilates pupils effectively while monitoring corneal health and vital signs. |  |
| 6 | **Speculum Selection** | Fails to select an appropriate size. | Selects an appropriate model. | Selects an appropriate model and size. | Selects an appropriate model and size; ensures proper eye opening for retinal visibility. |  |
| 7 | **Indentation** | Does not select appropriate equipment. | Selects appropriate equipment. | Selects and occasionally adjusts equipment. | Selects and adjusts equipment as needed. |  |
| 8 | **Laser Spot Size** | Neglects to select a proper size. | Selects an appropriate size with occasional assistance. | Independently selects an appropriate size. | Adjusts spot size based on the target area. |  |
| 9 | **Laser Settings (Power, Exposure, Interval)** | Fails to adjust settings appropriately. | Adjusts settings appropriately with some assistance. | Adjusts settings independently appropriately with minor modifications. | Adjusts settings precisely according to the target area. |  |
| 10 | **Laser Testing and Lining** | Fails to test laser strength or identify safe areas. | Tests laser strength in a safe area; requires assistance for ridge lining. | Tests laser strength in a safe area and partially completes lining. | Tests laser strength in a safe area and performs appropriate lining. |  |
| 11 | **Laser Spot Placement and Distribution** | Fails to understand proper spacing and critical areas. | Ensures appropriate placement and distribution in some areas. | Ensures appropriate placement and distribution in most areas. | Ensures appropriate placement and distribution throughout the avascular retina. |  |
| 12 | **Adequate Laser Spot Coverage** | Fails to identify required coverage. | Coverage is incomplete or excessive; requires significant correction. | Coverage is mostly complete with minor adjustments. | Achieves complete coverage, including the ridge and ora serrata. |  |
| 13 | **Cornea Erosion Awareness** | Fails to monitor hydration or handle instruments properly. | Monitors hydration and uses instruments appropriately during treatment. | Monitors hydration and examines the cornea post-treatment. | Monitors hydration, examines the cornea post-treatment, and provides post-treatment care. |  |

**Total Score:** ....................

| **No.** | **Global indices** | **Novice**  **(Score = 1)** | **Beginner**  **(Score = 2)** | **Advanced Beginner**  **(Score = 3)** | **Competent**  **(Score = 4)** | **Not Applicable**  **(Score = 0)** |
| --- | --- | --- | --- | --- | --- | --- |
| 1 | **Instrument Knowledge** | Cannot identify necessary instruments. | Identifies instruments but cannot use them appropriately, e.g., indentation tool or eye speculum. | Identifies and uses instruments appropriately, but without situational adjustments. | Identifies and uses instruments appropriately with situational adjustments. |  |
| 2 | **Procedure Flow** | Fails to maintain continuity. | Maintains treatment flow but requires significant time and guidance. | Maintains treatment flow with minimal interruptions. | Ensures smooth and continuous treatment flow. |  |
| 3 | **Laser Focus** | Does not maintain proper focus or inadvertently targets the macular area. | Maintains proper focus but frequently needs adjustments. | Maintains proper focus with occasional interruptions. | Maintains proper focus throughout the procedure. |  |
| 4 | **Continuous Laser Adjustment** | Does not adjust laser settings. | Adjusts initial settings and occasionally modifies during treatment. | Appropriately adjusts settings throughout treatment. | Continuously adjusts settings according to retinal conditions. |  |
| 5 | **Macular and Retinal Ridge Verification** | Fails to avoid targeting the macula or ridge. | Applies laser intermittently outside the retinal ridge and macula. | Consistently avoids targeting the retinal ridge and macula. | Safely avoids macula and ridge throughout the procedure. |  |

**Total Score:** ....................

**Laser Procedure Duration (based on recorded video):** .................. minutes.
**Proficiency Level:** (1) Novice (2) Beginner (3) Advanced Beginner (4) Competent.

**Comments and Recommendations:**

............................................................................................................................................................................................................................

............................................................................................................................................................................................................................

............................................................................................................................................................................................................................

............................................................................................................................................................................................................................
