# Supplementary material for: Deliberate practice for retinopathy of prematurity: Retinal laser training using schematic eyes in ophthalmology education
Source: PLoS One. 2025 May 29;20(5):e0323365. doi: 10.1371/journal.pone.0323365 (PMC12121759; doi:10.1371/journal.pone.0323365)
Supplement: S3 File — (DOCX) [file pone.0323365.s003.docx]

Institute: (1) PSU (2) KKU (3) CMU
ID: ____ ____

**Post-Research Survey**

Thank you for participating in the research project *Retinal Laser Training of ROP in Schematic Eyes*. With the conclusion of data collection, the research team kindly requests you to evaluate your confidence level after gaining various experiences during the study. Your feedback will contribute to the improvement of teaching and learning processes.

Please indicate how confident you feel about performing LIO in ROP on actual patients after completing each step.

Scoring system:
0 = Not confident at all
4 = Extremely confident

| No. | Question | Not Confident (0) | Slightly Confident (1) | Moderately Confident (2) | Very Confident (3) | Extremely Confident (4) |
| --- | --- | --- | --- | --- | --- | --- |
| 1 | After the LIO-ROP VDO study |  |  |  |  |  |
| 2 | After the MCQ test and comments from supervisors |  |  |  |  |  |
| 3 | After the first LIO-ROP in the schematic eye and assessment and feedback from the supervisors |  |  |  |  |  |
| 4 | After practicing LIO-ROP in the schematic eye |  |  |  |  |  |
| 5 | After the final LIO-ROP in the schematic eye and the assessment |  |  |  |  |  |

Suggestions for Improving Teaching and Learning

............................................................................................................................................................................................................................................................................................................
